# Outstanding Reviewers for *Nanoscale Advances* in 2024

**DOI:** 10.1039/d5na90045j

**Published:** 2025-07-22

## Abstract

We would like to take this opportunity to thank all of *Nanoscale Advances* reviewers for helping to preserve quality and integrity in the chemical science literature. We would also like to highlight the Outstanding Reviewers for *Nanoscale Advances* in 2024.

## Abstract 2

We would like to take this opportunity to highlight the Outstanding Reviewers for *Nanoscale Advances* in 2024, as selected by the editorial team for their significant contribution to the journal.

## Graphical abstract

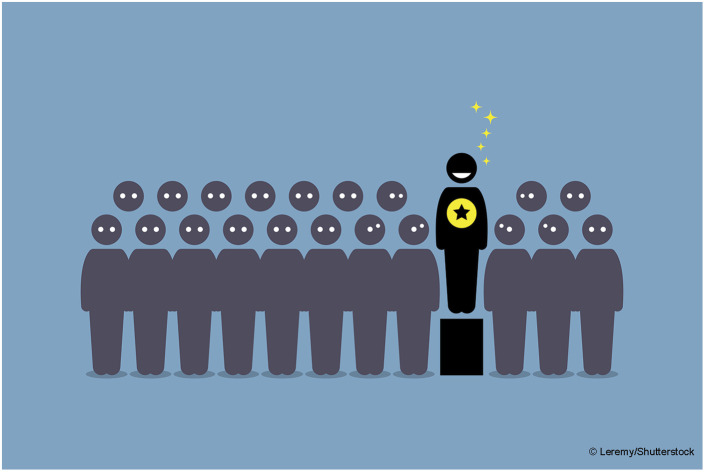

Each one of our outstanding peer reviewers has been carefully selected by our editorial team and includes active researchers who have made significant contributions to peer review and have gone above and beyond in their actions.

*“We extend our thanks to all of Nanoscale Advances reviewers for their invaluable contributions and are especially delighted to recognize this year’s Outstanding Reviewers for their exceptional dedication and support of the journal.”* – Professor Dirk M. Guldi, Editor-in-Chief

*“High-quality peer review, to a great extent, bridges the gap between initial manuscript level and journal’s publication standard, paving the way towards better content presentation and fresh knowledge spread for world-wide research communities. Therefore, we’re very delighted to recognize the Nanoscale Advances Outstanding Reviewers who have devoted continuous effort to manuscript review. Without their excellent professionalism and dedicated attitude, this journal’s publications could not be elevated. With concerted effort from all sides, we’re bound to make greater progress and generate more novel academic ideas on this promising open-access platform.”* – Professor Yue Zhang, Editor-in-Chief

In recognition of the varied contributions of our reviewer community, our Outstanding Reviewers from 2024 have been chosen based on several different measures, including the number, timeliness and quality of the reports completed over the last 12 months. In addition to reviewers who provided a high number of quality reports, we are pleased to highlight reviewers who provided exceptionally thorough and detailed reports, reviewers who provided clear and insightful adjudicative reports and reviewers who made additional efforts to aid authors in improving their manuscripts.

Dr Yee Sin Ang

Singapore University of Technology and Design

ORCID: 0000-0002-1637-1610

Dr Baoying Dai

Nanjing University of Posts and Telecommunications

ORCID: 0000-0003-1573-0279

Dr Pradip Das

University of Southampton

ORCID: 0000-0001-8898-331X

Dr Hideya Kawasaki

Kansai University

ORCID: 0000-0003-2713-2057

Dr Giovanni Marco Saladino

Stanford University

ORCID: 0000-0002-6854-1423

Professor Jessica Winter

The Ohio State University

ORCID: 0000-0002-5370-0137

Dr Hui Xu

Nanjing Tech University

ORCID: 0000-0002-9232-9625

Dr Ting Zhu

Yunnan Normal University

ORCID: 0000-0001-5567-3809

We would also like to thank the *Nanoscale Advances* Editorial Board and Advisory Board and the nanoscience community for their continued support of the journal, as authors, reviewers and readers.

We continue to work on improving the diversity of our reviewer pool to reflect the diversity of the communities that we serve.

Professor Dirk M. Guldi, Editor-in-Chief

Professor Yue Zhang, Editor-in-Chief

Dr Jeremy Allen, Executive Editor

